# Identification of a Novel Dihydroneopterin Aldolase as a Key Enzyme for Patulin Biodegradation in *Lactiplantibacillus plantarum* 6076

**DOI:** 10.3390/toxins18010048

**Published:** 2026-01-16

**Authors:** Yixiang Shi, Wenli Yang, Aidi Ding, Yuan Wang, Yu Wang, Qianqian Li

**Affiliations:** 1 College of Food Science and Engineering, Shanxi Agricultural University, Jinzhong 030801, China; syx0604@163.com (Y.S.); 20233695@stu.sxau.edu.cn (Y.W.); 2 Institute of Quality Standard and Testing Technology, Beijing Academy of Agriculture and Forestry Sciences, Beijing 100097, China; yangwenli@stumail.nwu.edu.cn (W.Y.); dingaidi2022@163.com (A.D.); 3 College of Life Science, Northwest University, Xi’an 710127, China

**Keywords:** patulin, biodegradation, proteomics, molecular docking, *Lactiplantibacillus plantarum*

## Abstract

Patulin (PAT) is a fatal mycotoxin that exerts serious threats to human and animal health. Biodegradation of PAT is considered to be one of the promising ways for controlling its contamination. In this study, *Lactiplantibacillus plantarum* 6076 (LP 6076) with reliable removal efficiency on PAT was screened out from three lactic acid bacteria (LAB) strains. It was found that the PAT was eliminated through degradation by LP 6076, and the intracellular proteins played a crucial role in PAT degradation with the induction of PAT. The proteomic analysis showed that the response of LP 6076 to PAT was by a concerted effort to repair DNA damage, in parallel to adaptive changes in cell wall biosynthesis and central metabolism. Eleven differentially expressed proteins with high fold changes were picked out and identified as PAT degradation candidate enzymes. The 3D structures of the candidate enzymes were predicted, and molecular docking between the enzymes and PAT was performed. Five enzymes, including Acetoin utilization AcuB protein (AU), GHKL domain-containing protein (GHLK), Dihydroneopterin aldolase (DA), YdeI/OmpD-associated family protein (YDEL), and Transcription regulator protein (TR), could dock with PAT with lower affinity and shorter distance. Through molecular docking analysis, DA was ultimately identified as a potential key degrading enzyme. The choice of DA was substantiated by its superior combination of strong binding affinity and a productive binding pose with PAT. VAL84 and GLN51 residues of DA were likely the active sites, forming four hydrogen bonds with PAT. Our study could accelerate the commercial application of biodegradation toward PAT decontamination.

## 1. Introduction

Fruits and their processed derivatives constitute a vital source of essential nutrients for human health. However, mycotoxin contamination has emerged as a significant determinant compromising their quality and safety. PAT, a polyketide lactone mycotoxin produced by various fungal species, including Penicillium and Aspergillus [1], exhibits notable stability under thermal and acidic conditions, coupled with high water solubility. This mycotoxin contaminates fresh fruits and their processed products throughout the agricultural supply chain, from raw materials to postharvest processing, thereby posing substantial public health risks and economic burdens. The World Health Organization (WHO) has established a maximum permissible limit of 50 µg/kg for PAT in apple juice and fruit-based beverages (WHO, 1995), a standard that has been adopted by over 100 countries and regions. Analytical surveillance data indicated that PAT levels in Japanese apple juice, Pakistani apples, Czech apple and pear products, Pakistani grapes, and Cameroonian maize significantly exceed regulatory thresholds [2]. PAT, a mycotoxin with demonstrated multi-organ toxicity, could induce a spectrum of toxicological effects ranging from acute and sub-acute poisoning to chronic intoxication, accompanied by cellular-level pathological alterations. Its toxicological profile encompassed teratogenic, carcinogenic, and mutagenic properties, thereby constituting a substantial threat to human health and safety [3]. These findings emphasized the imperative for implementing rigorous monitoring and control protocols throughout the fruit production and processing continuum.

The traditional technology mainly involves physical and chemical methods, but has some problems, such as incomplete degradation of patulin, high technical cost, and reduction in fruit quality. Biological degradation has gradually become a hot research topic in PAT control due to the advantages of being green, eco-friendly, and highly efficient. It is typically removed via desorption and degradation strategies.

Current strategies for PAT control primarily employ two mechanistic approaches: adsorption and degradation. The adsorption mechanism involves the interaction of PAT with functional groups (C=O, O–H, COO–, P–O–C, N–H, and –SH) present on surface-layer proteins and polysaccharides of certain microorganisms [4,5], but this method lacks specificity for PAT and may concomitantly adsorb essential nutrients, necessitating subsequent separation processes without achieving complete toxin removal. However, the degradation approach, wherein microorganisms produce specific detoxifying enzymes in response to toxin induction, can convert PAT into less toxic or non-toxic metabolites [3,6]. This method offers superior efficiency, thoroughness, and operational convenience, thus garnering increasing scientific attention.

Recent research results indicated that LAB may affect the pH of the medium, and may produce some unknown metabolites and have an impact on the quality of fruit juices and even foods [7,8]. Therefore, we are committed to exploring highly functional degrading enzymes for the degradation of PAT.

Recent advancements have identified thirteen PAT-degrading enzymes from microbial sources, including nucleotide diphosphate reductase, short-chain dehydrogenase/reductase, aldolase, and lipase, which function as lactone-degrading enzymes or reductases [9,10,11,12]. Additionally, certain transferases and oxidases have demonstrated PAT-degrading capabilities [13,14,15,16]. However, there were some restrictions of these enzymes for practical application, such as suboptimal degradation efficiency, insufficient stability, poor application performance, or lack of practical implementation, which were rooted in the unclear molecular mechanism between enzymes and PAT. Among them, only the degradation mechanism of CgSDR was illustrated [12].

In light of these limitations, the present study aimed to investigate the transformation mechanism of PAT by *Lactiplantibacillus plantarum* 6076, with particular focus on detoxification processes and degradation enzyme screening. This research employed proteomic analysis, protein structure simulation, and molecular docking techniques to identify and characterize the candidate PAT-degrading enzymes, thereby contributing to the development of more effective mycotoxin mitigation strategies.

## 2. Results and Discussion

### 2.1. Degradation of PAT by Three Viable and Heat-Killed LAB Strains

**Figure 1:**
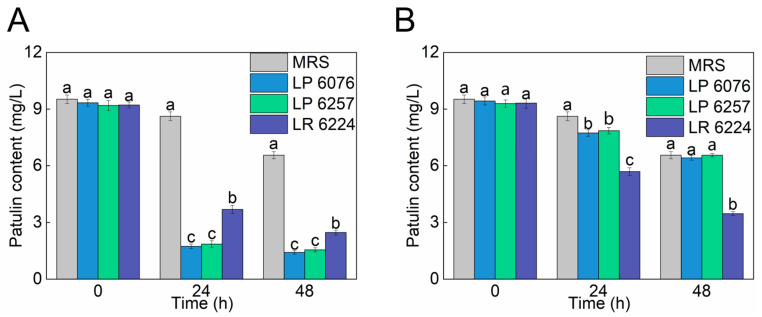
Effect of viable (**A**) and heat-inactivated (**B**) LP 6076, LP 6257, and LR 6224 on PAT reduction. All data are expressed as mean ± SD (*n* = 3). Different lowercase letters above the columns represented significant differences (*p* < 0.05) according to Duncan’s multiple range test.

Three LAB strains, LP 6076, LP 6257, and LR 6224, were cultured in MRS medium containing 10.9 mg/L PAT to evaluate their detoxification potential. During the 48 h incubation period, strain-specific degradation kinetics were observed through periodic PAT quantification (Figure 1A). LP 6076 exhibited the most pronounced degradation capacity, removing 84.1% of PAT within 24 h and achieving 87.2% elimination (final concentration 1.41 mg/L) by 48 h. This performance significantly surpassed the control group, which retained 8.62 mg/L PAT at the endpoint. LP 6257 also demonstrated a distinct degradation pattern, reducing PAT to 1.85 mg/L (83.1% removal) within the initial 24 h, followed by incremental degradation to 1.55 mg/L (85.8% total removal) by 48 h, while the LR 6224 displayed comparatively lower efficiency with 77.4% PAT reduction (2.46 mg/L residual) over the full 48 h period. The effects of heat-inactivated LP 6076, LP 6257, and LR 6224 on patulin reduction were also studied (Figure 1B). The inactivated LP 6076 and LP 6257 showed limited elimination capacity on PAT with the removal rates of 29.1% and 27.9%, respectively. However, LR 6224 could reduce PAT to the concentration of 5.69 mg/L (47.7% removal) after 24 h.

Based on the mode of patulin reduction, the treatment methods could be classified into two types: degradation and adsorption. The patulin degradation was due to the role of enzymes synthesized by the metabolism of each strain or patulin induction, while adsorption mainly depends on the cell microstructure and surface properties. The increased surface area and cell wall volume of the bacteria played key roles in patulin adsorption, and affected the main functional groups, C–O, –OH, and –NH, on the surface of microorganisms involved in adsorption [17,18,19]. Therefore, inactivated cells are mainly used for the adsorption of patulin, while active cells may have the function of adsorption and degradation. The effect of cell activity on the removal of patulin in phosphate-buffered saline was evaluated [20]. It displayed that 97.16% of patulin can be reduced by the viable *L. casei* YZU01 cells within 63 h, and only 20.64% of patulin bound onto the surface of heat-inactivated cells, which indicated that the inactivated cells adsorb a small amount of patulin, whereas the viable cells play key roles in patulin removal. Similarly, our research strongly suggested that active degradation metabolic processes, rather than passive adsorption, constituted the primary detoxification mechanism in LP 6076 and LP 6257, while both absorption and degradation were consistent in the PAT removal mechanism of LR 6224. During the same degradation period, the degradation effects of LP 6076 and LP 6257 were both higher than those of LR 6224. There was no significant difference in the degradation effects between LP 6076 and LP 6257, but the growth rate of LP 6076 was twice as fast as that of LP 6257. Therefore, the LP 6076 was the most suitable LAB to reduce patulin and was used for subsequent analysis.

### 2.2. PAT Degradation by Extracellular or Intracellular Enzymes of LP 6076

**Figure 2:**
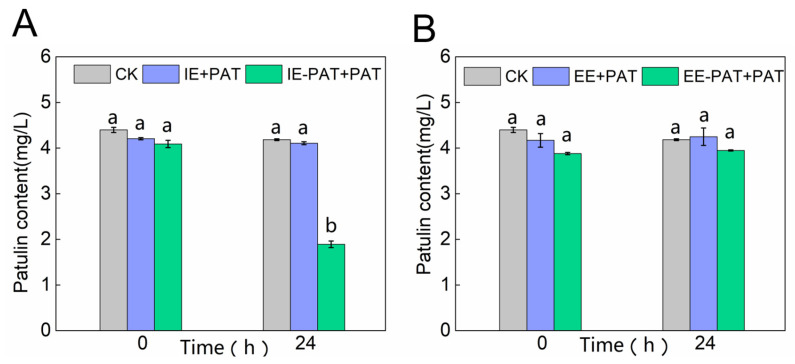
PAT degradation by extracellular enzymes of LP 6076 (**A**) or intracellular enzymes (**B**) CK represented the control groups containing only patulin. The degradation activity was assessed by incubating PAT with extracellular enzyme (EE) or intracellular enzyme (IE) fractions. The enzyme fractions were either derived from untreated cells (EE+PAT and IE+PAT) or from cells pre-treated with patulin (EE-PAT+PAT and IE-PAT+PAT). All data are expressed as mean ± SD (*n* = 3); different lowercase letters among one time point represented significant differences (*p* < 0.05) according to Duncan’s multiple range test.

The PAT degradation effects by the extracellular enzymes of LP 6076 (EE+PAT) and extracellular enzymes pretreated with PAT (EE-PAT+PAT) were demonstrated (Figure 2A). The residual PAT concentrations of EE+PAT and EE-PAT+PAT groups were similar to the control group after 24 h incubation, indicating that the extracellular enzymes of LP 6076 had almost no degradation effect on patulin, regardless of whether pretreated with PAT or not. Subsequently, the intracellular enzymes of LP 6076 were obtained by ultrasonication and filtration. The PAT degradation effects by the intracellular enzymes (IE+PAT) and intracellular enzymes pretreated with PAT (IE-PAT+PAT) were determined (Figure 2B). There was no significant difference between the groups of IE+PAT and the control group after 24 h incubation, while 53.8% of PAT was degraded by intracellular enzymes of LP 6076 pretreated by PAT. These findings demonstrated that the PAT degradation of LP 6076 was an enzyme-mediated mechanism, the degradation activity resulted from the production of specific PAT-metabolizing enzymes by LP 6076, and not from the action of constitutive extracellular enzymes or non-specific physical adsorption. Similarly, PAT degradation by *Candida guilliermondii* was found to be dependent on the yeast cell viability and to occur mainly inside the cells [21]. Furthermore, intracellular enzymes produced by *Pichia caribbica* [14], *Meyerozyma guilliermondii* [22], *Pichia guilliermondii* strain S15-8 [23], *Saccharomyces cerevisiae* [24], and *Bacillus mojavensis* YL-RY0310 [25] showed the ability to degrade PAT. In contrast to our findings, PAT degradation has been reported to be mediated by extracellular enzymes of *Lactobacillus casei* YZU01 [20] and *Ralstonia* [13]. In addition, the ability of intracellular material to degrade PAT was more pronounced when LP 6076 was co-cultured with PAT for 24 h compared to the control group, suggesting that the ability of LP 6076 to degrade PAT can be enhanced by PAT induction. Analogously, an acceleration of PAT degradation was observed by Ianiri [6] when crude intracellular protein extracts of *Sporobolomyces* sp were prepared from cells pretreated with PAT. Zhong [26] found that intracellular enzymes of the *Saccharomyces cerevisiae* S288C were key factors in PAT degradation, and these degradation-active intracellular enzymes in *S. cerevisiae* were obtained after PAT induction. *Pseudomonas aeruginosa* TF-06 could degrade patulin into non-cytotoxic E/Z-ascladiol mainly by the activity of intracellular enzymes, which was induced by PAT [27]. Our studies were consistent with these results that PAT degradation was relevant to the intracellular enzymes produced by bacteria or yeast in response to the induction of patulin. This study provides a critical theoretical foundation for optimizing microbial detoxification strategies.

### 2.3. Molecular Response of LP6076 to PAT

#### 2.3.1. Identification of Differentially Expressed Proteins

**Figure 3:**
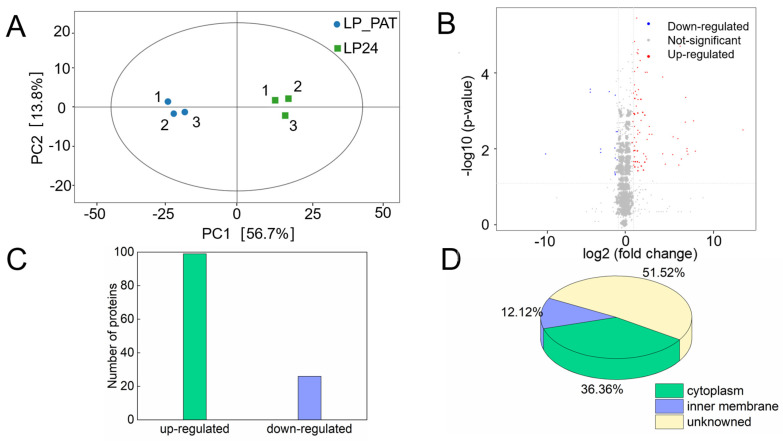
Differentially expressed proteins of LP 6076 under PAT stress: (**A**) PCA. (**B**) Correlation volcano map of samples. (**C**) The number of up-regulated and down-regulated proteins. (**D**) Subcellular location analysis pie chart of samples. LP_PAT represented LP 6076 incubated with PAT for 24 h. LP24 represented LP 6076 incubated for 24 h without PAT.

To investigate the metabolic response of LP 6076 to PAT degradation, DIA quantitative proteomics was used to detect the differential expression proteins in LP 6076 under PAT stress. Principal component analysis (PCA) (Figure 3A) was performed to assess sample homogeneity in the proteomic study. The first two principal components (PC1 and PC2) cumulatively accounted for 72.4% of total variance (PC1: 58.2%; PC2: 14.2%), indicating sufficient data dimensionality reduction for robust pattern recognition. Samples among the three repeats exhibited tight clustering within the PCA score plot (average inter-sample Euclidean distance = 1.83 ± 0.41), demonstrating high biological reproducibility and meeting the prerequisite for comparative proteomic analysis. A total of 2301 proteins were identified in this study, 99 up-regulated proteins and 26 down-regulated proteins were identified in LP 6076 incubated with PAT for 24 h compared with LP 6076 incubated for 24 h without PAT (Figure 3B,C, Fold change ≤ 0.5 and *p-*value < 0.05). Subcellular organelles are important sites of protein functions, and the analysis of the localization of subcellular proteins in our study contributed to a better understanding of the role of differential expression proteins in the cell, which showed that the majority of the differential expression proteins were located in the cytoplasm (36.36%), followed by the inner membrane (12.12%), others are secreted (Figure 3D). The degradation effect of PAT by LP 6076 is mainly the result of intracellular enzymes, which is consistent with the identified localization of differential expression proteins. However, there were multiple intracellular enzymes in LP 6076, and the key enzymes that could degrade PAT remained unclear.

#### 2.3.2. Functional Classification of Differentially Expressed Proteins

**Figure 4:**
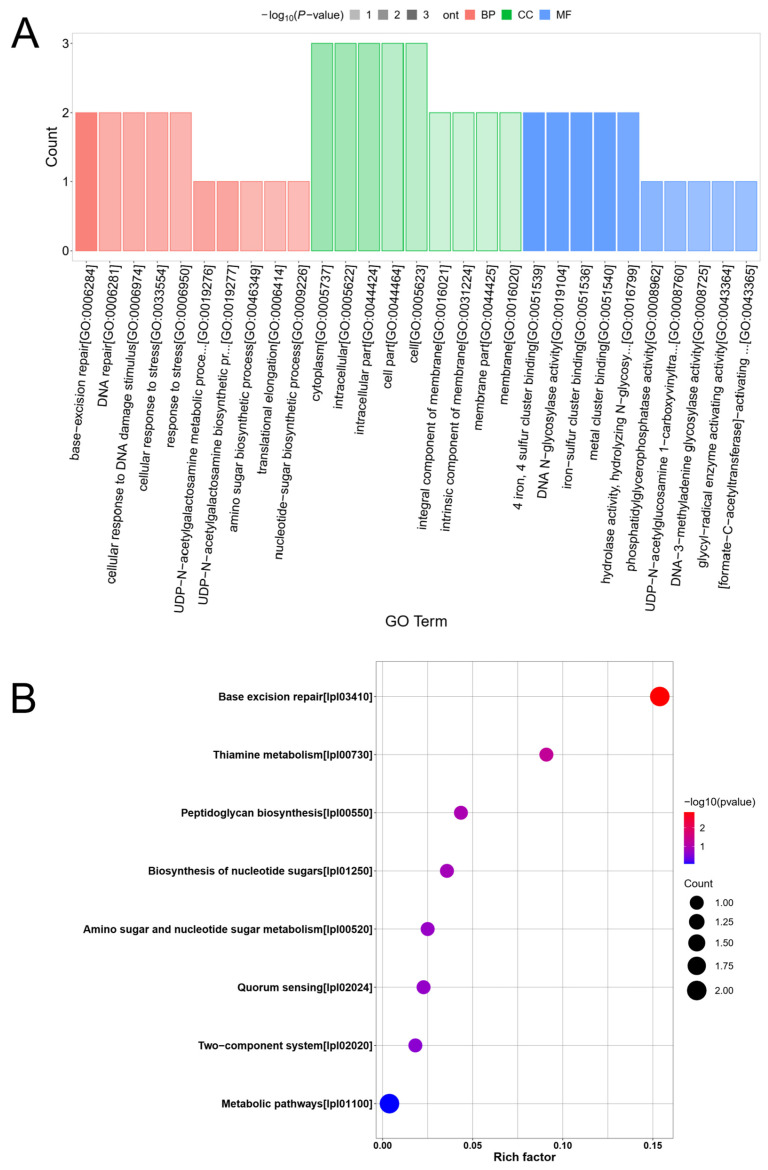
Functional classification of differentially expressed proteins: (**A**) Gene ontology (GO) analysis. (**B**) The KEGG pathway enrichment analysis. BP, biological processes. CC, cellular component. MF, molecular function.

To further evaluate the function, location, and biological pathways of differentially expressed proteins in LP 6076 after PAT stress, the GO and KEGG enrichment analyses were performed to predict the cellular defense mechanisms. The GO enrichment analysis revealed significant alterations in cellular processes under PAT stress (Figure 4A). In the biological processes (BP) category, terms related to DNA damage repair were most prominently enriched, including base-excision repair (GO:0006284), DNA repair (GO:0006281), and cellular response to DNA damage stimulus (GO:0006974). Metabolic processes, such as nucleotide-sugar biosynthetic process (GO:0009226) and UDP-N-acetylgalactosamine biosynthetic process (GO:0019277), were also significantly up-regulated. In the cellular component (CC), proteins were primarily localized to the cytoplasm (GO:0005737) and integral membrane components (GO:0016021). Molecular function (MF) analysis highlighted enrichment in iron–sulfur cluster binding (GO:0051536) and DNA glycosylase activity (GO:0019104), indicating adaptive functional shifts.

The KEGG pathway enrichment analysis of the differentially expressed proteins in LP 6076 under PAT stress revealed significant alterations in key metabolic and repair pathways (Figure 4B). The base excision repair pathway exhibited the most pronounced enrichment, as indicated by the highest rich factor and a strong statistical significance (−log10(*p*-value) > 2). Other pathways, including thiamine metabolism and peptidoglycan biosynthesis, were also significantly enriched, though to a lesser extent.

In this study, the integrated GO and KEGG analyses demonstrated that LP 6076 mounted a coordinated, multi-faceted defense strategy against PAT-induced stress. The pronounced enrichment of the base excision repair pathway and related DNA repair functions in both analyses indicated that PAT exerted genotoxic effects, likely causing DNA base damage. Yang [28] demonstrated that the proteins involved in biological processes such as DNA damage repair and base excision repair were significantly up-regulated in LP 6076 under PAT stimulation. The up-regulation of DNA glycosylases and other base excision repair components represented a primary cellular defense mechanism to maintain genomic integrity. Simultaneously, metabolic shifts were evident through the enrichment of nucleotide sugar and amino sugar biosynthesis pathways. These changes suggest a restructuring of cell wall and membrane components, possibly to enhance structural integrity under toxin-induced membrane perturbation. The involvement of peptidoglycan and nucleotide sugar metabolic pathways may reflect an adaptive response to maintain cellular rigidity and signal transduction capacity. In conclusion, the proteomic response of LP 6076 to PAT was characterized by a prioritized effort to repair DNA damage, coupled with adaptive changes in cell wall biosynthesis and central metabolism. These findings provide a comprehensive view of bacterial adaptation mechanisms to patulin stress, revealing key molecular targets for potential intervention strategies.

### 2.4. Mining of PAT Degradation Enzymes in LP6076

In this study, from the pool of differentially expressed proteins, we identified 99 up-regulated proteins with a fold change (FC) greater than two. Among these, the top 11 proteins exhibiting higher FC values (FC > 198) were considered as candidate enzymes for PAT degradation (Table 1). The protein sequences of these 11 candidates were retrieved from the UniProt database. These 11 proteins were ultimately selected for structural prediction using AlphaFold. For each protein, AlphaFold generated four theoretical three-dimensional (3D) models, each accompanied by distinct confidence metrics, including the confidence score (C-score), template modeling score (TM-score), and global model quality estimation (GMQE), which collectively assess the reliability and accuracy of the predicted structures. The most plausible model was selected depending on the higher GMQE value (0.92, 0.68, 0.83, 0.71, and 0.62). Subsequently, molecular docking simulations between each protein and PAT were performed using AutoDockTools. Prior to docking, the protein and ligand files, originally in PDB format, were converted into PDBQT format to incorporate atomic properties such as atom types, bond types, and partial charges. Following the docking process, proteins exhibiting positive binding energies, indicating a requirement for external energy to facilitate interactions with PAT, were excluded from the candidate list. Consequently, proteins demonstrating lower binding energies were retained as potential candidates for PAT degradation.

**Figure 5:**
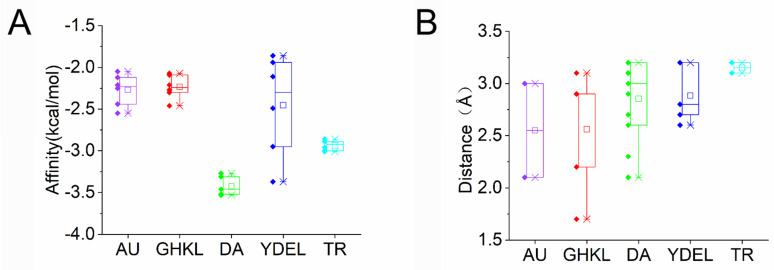
(**A**) calculated distances between catalytically important atoms of enzymes and labile groups of PAT. (**B**) calculated distances between catalytically important atoms of enzymes and labile groups of PAT. Designation: □—mean value; × — 1st and 99th quantile; ◆—value of individual pose. AU, Acetoin utilization AcuB protein; GHKL, GHKL domain-containing protein; DA, Dihydroneopterin aldolase; YDEL, YdeI/OmpD-associated family protein; TR, Transcription regulator protein.

**Figure 6:**
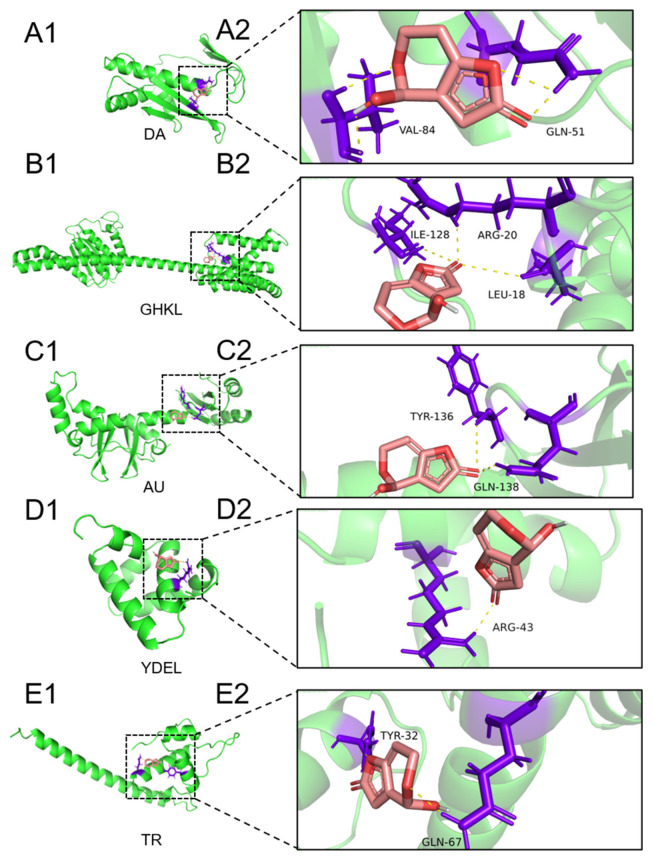
Molecular docking analysis of the binding capacity of PAT with potential enzymes. (**A1**–**E1**) were the results of the 3D docking of different enzymes with PAT, respectively, and (**A2**–**E2**) were the results of the PAT binding pattern with protein residue sites. The red stick represented PAT, and the yellow dotted line represented the hydrogen bond.

The final selection included five proteins: Acetoin utilization AcuB protein (AU), GHKL domain-containing protein (GHLK), Dihydroneopterin aldolase (DA), YdeI/OmpD-associated family protein (YDEL), and Transcription regulator protein (TR). Among these, DA exhibited the most favorable binding energy (ΔG = −3.53 kcal/mol) when docked with PAT (Figure 5A). Key interactions were observed between PAT and specific amino acid residues within the active site of DA. Notably, VAL84 and GLN51 were predicted to act as hydrogen bond donors, forming hydrogen bonds with the oxygen atom of PAT, thereby stabilizing its conformation at the active center (Figure 6A1,A2). The distances between PAT and these residues were calculated as 3.2 Å and 2.1 Å, respectively (Figure 5B). Similarly, GHLK demonstrated a binding energy of −2.46 kcal/mol with PAT, stabilized by hydrogen bonds involving residues ARG20, LEU18, and ILE128, with respective distances of 2.9 Å, 3.1 Å, and 1.7 Å (Figure 5A,B and Figure 6B1,B2). For AU, a binding energy of −2.55 kcal/mol was observed, with residues TYR136 and GLN138 forming hydrogen bonds with PAT at distances of 3.0 Å and 2.2 Å, respectively (Figure 5A,B and Figure 6C1,C2). YDEL exhibited a binding energy of −3.37 kcal/mol, with ARG43 acting as a hydrogen bond donor at a distance of 3.2 Å from PAT (Figure 5A,B and Figure 6D1,D2). Finally, TR displayed a binding energy of −3.01 kcal/mol, with residues TYR32 and GLN67 forming hydrogen bonds with PAT at distances of 3.2 Å and 3.1 Å, respectively (Figure 5A,B and Figure 6E1,E2). This study integrated proteomic discovery with computational structural biology to pinpoint a leading candidate enzyme for PAT degradation. The molecular docking results moved beyond the initial list of stress-responsive proteins and provided a mechanistic rationale for selecting the DA enzyme for further validation.

The choice of DA was substantiated by its superior combination of strong binding affinity and a productive binding pose. The significantly lower binding energy of DA compared to other candidates indicated a thermodynamically more favorable and stable enzyme–substrate complex formation. The visualization suggested that the binding pocket of GHKL, involving residues like ILE128 and LEU18, might be suboptimal. The presence of a bulky hydrophobic residue like ILE128 could lead to a less complementary fit with the planar and polar regions of the patulin molecule, resulting in fewer van der Waals contacts or hydrogen bonds. This imperfect fit and the consequent weaker binding energy made GHKL a less attractive candidate than DA. The AU group, on the other hand, exhibited the weakest binding and the longest intermolecular distances, suggesting only a weak, non-specific association with PAT, rendering it an unsuitable candidate for efficient degradation. Therefore, it is likely that DA was the enzyme that degraded PAT in LP6076. The functional significance of aldolase in PAT degradation warranted particular attention, such as the aldolase identified from *Rhodotorula mucilaginosa* [10]. As a key enzyme in biotoxin metabolism, aldolase catalyzes the conversion of toxic aldehydes to less harmful alcohols, thereby mitigating oxidative stress and cellular damage. Our findings suggested that LP6076’s aldolase system may represent an evolutionary adaptation for mycotoxin resistance, with important implications for food safety applications.

## 3. Conclusions and Perspectives

Biological degradation based on microbial enzymes is considered to be one of the best alternatives for controlling PAT contamination. However, few enzymes have been reported to have PAT degradation ability until now. In this study, we identified a LAB strain LP 6076 that could remove 84.1% of PAT within 24 h and achieve 87.2% elimination effectively, and demonstrated that PAT degradation by LP 6076 was attributed to its intracellular enzymes mediated by PAT induction. The proteomic analysis showed that the response of LP 6076 to PAT was by a concerted effort to repair DNA damage, in parallel to adaptive changes in cell wall biosynthesis and central metabolism. We identified a novel PAT-degrading aldolase, designated DA, from the LP6076. Furthermore, structure prediction and molecular docking analyses suggested that GLN51 and VAL84 were likely crucial residues of DA for the degradation reaction. The newly identified DA enzyme holds significant promise for developing practical enzymatic preparations for food safety. Future study will focus on heterologous expression and production of DA to facilitate its application in fruit. It is crucial to characterize the enzyme’s kinetic properties, optimal working conditions (pH and temperature), and degradation products to ensure its efficacy and safety in food products. Moreover, site-directed mutagenesis of the key residues (GLN51 and VAL84) could be employed to enhance the enzyme’s activity and stability. Ultimately, engineering DA into immobilized enzymes could provide robust and scalable solutions for PAT detoxification in apple juice and other susceptible food products, paving the way for greener and more specific food preservation technologies. These findings provided new insights for exploring PAT degradation enzymes and developing enzymic formulations for PAT detoxification in the food industry.

## 4. Materials and Methods

### 4.1. LAB Strains and Culture Conditions

Three strains of lactic acid bacteria (LAB), *Lactiplantibacillus plantarum* 6076 (LP 6076), *Lactiplantibacillus plantarum* 6257 (LP 6257), *Lactobacillus rhamnosus* 6224 (LR 6224), were obtained from the China Center of Industrial Culture Collection (CICC, Beijing, China). The strains were preserved in 15% (*v*/*v*) glycerol solution and stored at −80 °C until use. Prior to experimental procedures, the LAB strains were activated by culturing in de Man, Rogosa, and Sharpe (MRS) (Solabrio, Beijing, China) broth at 37 °C, 180 rpm for 6.5 h under aerobic conditions. The activated cultures were used for subsequent experiments.

### 4.2. Screening LAB Strains for PAT Degradation

The activated LAB strains (1 mL) were inoculated into 100 mL of MRS broth and incubated at 37 °C, 180 rpm for 6.5 h under aerobic conditions. The viable experimental group took 10 mL of LAB strains for vortex washing with sterile Milli-Q water and suspended it in 10 mL of phosphate-buffered saline (PBS), and it was prepared by adding 10 mg/L PAT. The inactivated experimental group was autoclaved (121 °C, 0.1 MPa for 15 min). After inactivation, the cells were recollected by centrifugation (10,000× *g*, 4 °C, 10 min) and mixed with 10 mL PBS containing 10 mg/L PAT. A control group was prepared by adding 10 mg/L PAT to 10 mL PBS without LAB inoculation. All the samples were incubated at 37 °C. PAT was extracted and quantified at 0, 24, and 48 h of incubation. For extraction, 1 mL of culture was centrifuged at 10,000× *g* for 5 min at 4 °C, and the supernatant was collected. The supernatant was then filtered through a 0.22 μm microporous membrane. PAT analysis was conducted using a high-performance liquid chromatography (HPLC) system (Agilent, 1260 Infinity, Walter Blon, Germany) equipped with a reversed-phase C18 column (Agilent, 5 μm, 250 × 4.6 mm, Germany). The limit of detection (LOD) and limit of quantitation (LOQ) for the HPLC method were determined to be 0.04 mg/L and 0.13 mg/L, respectively. The mobile phase consisted of Milli-Q water and acetonitrile (9:1, *v*/*v*), delivered at a flow rate of 1 mL/min. Detection was performed using a variable wavelength detector (Agilent, Beijing, China) set at 276 nm. The 10 μL aliquot of each filtered sample was automatically injected for analysis. Quantification was achieved by constructing a standard curve through linear regression analysis, correlating the peak area with known PAT concentrations (0.1–10 mg/L).

### 4.3. Effect of the Extracellular Enzymes of LP 6076 on PAT Degradation

Two groups were set as follows: group 1, 40 μL of the activated LP 6076 and 1 mL of MRS liquid media; group 2, 40 μL of the activated LP 6076 and 1 mL of MRS liquid media and PAT (final concentration of 5 mg/L). The samples were incubated at 37 °C. The LP 6076 extracellular enzymes and extracellular enzymes stimulated by PAT were obtained by centrifuging at 10,000× *g* for 5 min at 4 °C and filtering from the culture medium through a 0.22 μm microporous membrane at 24 h post incubation. PAT (final concentration of 5 mg/L) was added to the culture medium of group 1 (EE+PAT), 2 (EE-PAT+PAT), and an equal amount of PAT was added to PBS as a control (CK). All the samples were incubated at 37 °C and collected at 0 and 24 h after the last incubation for the detection of PAT content.

### 4.4. Effect of Intracellular Enzymes of LP 6076 on PAT Degradation

The activated LP 6076 (80 μL) was inoculated into 2 mL MRS liquid media without or with PAT (5 mg/L) and incubated at 37 °C for 24 h. The LP 6076 cells were collected, transferred into a 10 mL tube, and centrifuged at 10,000 rpm and 4 °C for 10 min. The pellets were collected and washed twice with 5 mL PBS. The cells were then resuspended in 1 mL PBS. The LP 6076 cells were broken by sonication at 300 W, with an interval of 2 s, work of 4 s, and repetition of 60 times. The supernatant was collected by centrifugation at 10,000 rpm and 4 °C for 5 min, and was considered as LP 6076 intracellular enzymes and intracellular enzymes stimulated by PAT. Afterwards, PAT was added to intracellular enzymes that were extracted from LP 6076 amended with (IE-PAT+PAT) or without PAT (IE+PAT), with the final concentration of 5 mg/L. The sample containing PBS and PAT (final concentration of 5 mg/L) was used as the control. All the samples were incubated at 37 °C and collected at 0 and 24 h for PAT detection.

### 4.5. Proteomic Technology and Analysis

In this study, two distinct sample groups were prepared for proteomic analysis, each comprising three biological replicates to ensure statistical robustness. The experimental groups were defined as follows: (1) LP 6076 culture without PAT supplementation (designated as LP24), incubated under controlled conditions (180 rpm, 37 °C for 24 h); and (2) LP 6076 culture supplemented with 5 mg/L PAT (designated as LP_PAT), maintained under identical incubation parameters. Both groups were processed in triplicate to account for biological variability.

For protein extraction, one-third of each sample aliquot was transferred to a sterile microcentrifuge tube (Solabrio, Beijing, China) and homogenized with 200 µL of RIPA lysis buffer. Mechanical disruption was achieved through low-temperature grinding utilizing stainless steel beads, with operational parameters set at 35 Hz frequency for 4 min at 12,000 rpm. Following homogenization, the samples were centrifuged at 4 °C for 10 min (12,000 rpm). The resultant lysate was subjected to sonication in an ice-water bath for 20 min to ensure complete cellular lysis. A subsequent centrifugation step was performed under identical conditions, after which the clarified supernatant was collected for downstream processing.

Protein quantification was performed using the bicinchoninic acid (BCA) assay, followed by acetone precipitation to concentrate the protein fraction. The proteins were then subjected to reduction and alkylation to prepare for enzymatic digestion. Trypsin digestion was carried out to generate peptides, which were subsequently desalted prior to mass spectrometric analysis. Liquid chromatography-tandem mass spectrometry (LC-MS/MS) analysis was performed using data-independent acquisition (DIA) mode on a Bruker timsTOF Pro2 (Bruker, Beijing, China) instrument, employing a nano-LC gradient separation. Raw mass spectrometry data were processed using Spectronaut software (version 18.2.230802.50606; Biognosys AG) for qualitative protein identification and quantification. This analytical pipeline ensured comprehensive proteomic profiling while maintaining rigorous quality control throughout the experimental workflow.

### 4.6. Molecular Docking

The computational methodology for enzyme–substrate interaction analysis was adapted from previously established protocols [29]. The initial structural data for PAT were retrieved from the PubChem database in SDF format and subsequently converted to PDB format using the Open Babel (version 3.1.1) software. Structural preparation for molecular docking was performed using AutoDockTools [30] (integrated within MGLTools version 1.5.6), wherein the PDB files were converted to PDBQT format, incorporating necessary polar hydrogens and Gasteiger charges.

To identify potential PAT-degrading enzymes, differential protein expression analysis was conducted based on proteomic data, with particular emphasis on proteins exhibiting the highest fold-change values. These candidate proteins were cross-referenced against the UniProt database to identify putative enzymatic candidates. The corresponding amino acid sequences of these candidate enzymes were retrieved from UniProt and subjected to structure prediction using AlphaFold (version 2).

Molecular docking simulations were performed using AutoDock software (version 1.5.7) to investigate enzyme–substrate interactions. The grid box dimensions were optimized to encompass the entire enzyme structure with sufficient margin, centered on the enzyme’s mass centroid. Docking parameters were maintained at default settings, with the enzyme (receptor) treated as a rigid body and PAT (ligand) allowed full torsional flexibility. From the docking simulations, the six most energetically favorable binding poses were selected for further analysis.

Catalytically relevant interactions were characterized by measuring interatomic distances between key residues in the enzyme’s active site and reactive centers of the PAT molecule. Structural visualization and distance measurements were conducted using PyMOL Molecular Graphics System (version 3.1.1), enabling detailed analysis of potential catalytic mechanisms and substrate binding orientations. This comprehensive computational approach provided molecular-level insights into enzyme–substrate interactions and potential PAT degradation pathways.

### 4.7. Statistical Analysis

All data were expressed as mean ± SD. Data were analyzed by one-way ANOVA followed by Duncan’s multiple range test for post hoc comparisons. Different lowercase letters indicate significant differences (*p* < 0.05).

## Figures and Tables

**Table 1:** The selected proteins and their descriptions.

Accession	Protein Name	*p*-Value	Fold Change (FC)
A0A0G9FAZ5	Acetoin utilization acuB protein	0.004	31,374.29
A0A0G9FAL3	GHKL domain-containing protein	0.183	529.54
A0A0G9FCJ9	YdeI/OmpD-associated family protein	0.016	520.25
A0A0G9GHX1	UPF0346 protein	0.002	443.85
A0A0L7Y4X5	Dihydroneopterin aldolase	0.102	279.77
A0A0G9GPC3	Uncharacterized protein	0.013	258.41
A0A162GIQ5	Transcription regulator	0.016	245.42
B3Y991	Transposase	0.054	229.42
A0A0R2GG82	Nisin resistance protein	0.042	207.99
A0A0R2GES8	Acetyltransferase	0.007	204.78
A0A165EZP0	Uncharacterized protein	0.186	202.99

## Data Availability

The original contributions presented in this study are included in the article. Further inquiries can be directed to the corresponding authors.
